# Determination of antimicrobial effect of protamine by transmission electron microscopy and SDS PAGE on *Pseudomonas aeruginosa *isolates from diabetic foot infection 

**DOI:** 10.22038/ijbms.2019.32414.7989

**Published:** 2019-07

**Authors:** Mubashar Aziz, Rafael Garduno, Zulfiqar Ali Mirani, Rakhshanda Baqai, Ahsan Sattar Sheikh, Humera Nazir, Yasir Raza, Mazhar Ayaz, Shahana Urooj Kazmi

**Affiliations:** 1 Department of Pathobiology, Bahauddin Zakariya University, Multan, Pakistan; 2 Department of Microbiology, University of Karachi, Karachi, Pakistan; 3 Department of Microbiology, Dalhousie University, Halifax, Canada,; 4 PCSIR Laboratories Complex, Karachi, Pakistan; 5 Departmet of Clinical Microbiology & Immunology, Dadabhoy Institute of Higher Education, Karachi, Pakistan; 6 Institute of Food Science and Nutrition, Bahauddin Zakariya University, Multan, Pakistan; 7 Islamic International University, Islamabad

**Keywords:** Diabetic foot, Pseudomonas aeruginosa, Protamine, Transmission electron – Microscopy, Polyacrylamide gel

## Abstract

**Objective(s):**

Diabetic foot infection is one of the major complications of diabetes leading to lower limb amputations. Isolation and identification of bacteria causing diabetic foot infection, determination of antibiotic resistance, antimicrobial potential of protamine by electron microscopy and SDS-PAGE analysis, arethe aims of this study.

**Materials and Methods:**

285 pus samples from diabetic foot infection patients were collected from different hospitals of Karachi and Capital Health Hospital, Halifax, Canada. Clinical history of each patient was recorded. Bacterial isolates were cultured on appropriate media; identification was done by morphology, cultural and biochemical tests. Effect of protamine against multi drug resistant strains of *Pseudomona aeruginosa *was checked by minimum inhibitory concentration in 96 well micro-titer plates. The isolates were grown in bactericidal concentration of protamine on plates to isolate mutants. Effect of protamine on protein expression was checked by SDS- PAGE and ultra-structural morphological changes by transmission electron microscopy.

**Results:**

Results indicated prevalence of foot infection as 92% in diabetic patients. Major bacterial isolates were *Staphylococcus aureus* 65 (23%), *P. aeruginosa *80 (28.1%), *Klebsiella *spp. 37 (13%), *Proteus mirabilis* 79 (27.7%), and *Escherichia coli *24 (12%). These isolates were highly resistant to different antibiotics. MIC value of protamine was 500 µg/ml against *P. aeruginosa*. SDS-PAGE analysis revealed that protamine can suppress expression of various virulence proteins and electron micrographs indicated condensation of cytoplasm and accumulation of protamine in cytoplasm without damaging the cell membrane.

**Conclusion:**

*P*. *aeruginosa* and *S. aureus *were the major isolates expressing multi-drug resistance and protamine sulfate represented good antimicrobial potential.

## Introduction

Diabetic foot infection is one of the foremost complications of diabetes leading to hospitalization and lower limb amputations (1). These infections are usually poly-microbial (2). Severe tissue damage in diabetic foot ulcers is frequently associated with *Pseudomona aeruginosa *wound invasion. A major problem reported with *P. aeruginosa *infection is its high degree of resistance to some broad-spectrum antibiotics which make it to grow in different challenging environments (3, 4). Antibiotic resistance warrants to finding alternative therapies which not only directly target bacterial viability but also can counter pathogen’s ability to damage the host, significantly. Such alternative therapies can reduce the global burden of antibiotic resistant strains for available antimicrobial therapy (5).

Protamine sulfate is a well-known cationic polypeptide (CAP) derived from salmon fish sperm. Bactericidal effect of protamine is well known (6). Several CAPs are thought to express antimicrobial activity due to their pore forming ability that leads to destabilization of cytoplasmic membrane of susceptible bacteria resulting into cell lysis (7, 8). Two distinct properties are usually present in cationic antimicrobial peptides. These are amphipathic with hydrophobic and hydrophilic domains and are polycationic in nature (9-11). Peptides may interact with teichoic acids in Gram-positive bacteria and lipopolysaccharide in Gram- negative bacteria (9). Antimicrobial potential and its effect on cell morphology have been well investigated and reported across the globe (12-18). The present study is designed to reconnoiter the antimicrobial potential of protamine on multi drug resistant (MDR) *P. aeruginosa *isolated from diabetic foot infections. Moreover, transmission electron microscopy would reveal morphological effect produced by protamine on bacterial structure(s) of clinical isolate of *P. aeruginosa*. Furthermore, SDS-PAGE analysis has also been done to investigate the changes produced by protamine sulphate in the protein profile of non-stressed and stressed *P. aeruginosa* isolated from diabetic foot infection. 

## Materials and Methods

***Ethical certificate***

The present study was approved by the Ethical Review Board, University of Karachi, Pakistan.

***Epidemiology of diabetic foot patients***

A total of 350 diabetic patients with foot infection in the age groups of 30 to 80 years were included in the study. Both outdoor and hospitalized patients from different hospitals of Karachi, Pakistan were included. Clinical history was collected from all patients on a specially designed proforma along with the consent form to collect pus sample. 

***Isolation, identification and antibiotic sensitivity of isolates***

Pus samples were collected from ulcers in each case with Amies Transport medium swabs (19). All specimens were streaked on Blood agar (Oxide, UK), MacConkey’s agar (Oxide, UK) and Chocolate agar (Oxoide, UK) for medium isolation of aerobic and anaerobic microorganisms. Plates were incubated at 37 ^°^C in incubator (Memmert, Germany) for 24 hr. The bacterial isolates were identified by standard biochemical methods (20). Antibiotic resistance on diabetic foot isolates was performed on Iso Sensitivity agar plates (Oxoid, UK) by Kirby Bauer’s Disc Diffusion Method as per Clinical Laboratory Standards Institute guidelines (CLSI, 2010). *P. aeruginosa* isolates were characterized as MDR as defined by European Center for Disease Prevention ( non-susceptibility of pathogen to at least one agent in ≥3 antimicrobial categories).

***Determination of minimum inhibitory concentration (MIC) of protamine***

MIC was determined for protamine (ICN Biomedical, USA) against 20 MDR *P. aeruginosa *isolates, as reported previously (21). The cultures were grown in Nutrient broth for 24 hr at 37 ^°^C. Protamine mutant strains were incubated at room temperature for 5 days. MIC was determined by using 96 wells flat bottom micro-titer plates (polystyrene,Micro Test 96^TM^, BD, USA). 

***Antimicrobial stress effects on expression of outer membrane proteins in P. aeruginosa***

Effect of antimicrobial potential of protamine sulfate against outer membrane proteins expressed in *P. aeruginosa* was performed as reported previously with some modifications (22). Isolated colonies of *P. aeruginosa* were inoculated into 2 ml Nutrient broth and incubated at 37 ^°^C for 24 hr. Next day, this 2 ml culture was added into 48 ml Nutrient broth on a shaking incubator at 200 RPM for 24 hr at 37 ^°^C in the presence and absence of protamine sulfate in sub lethal dose (100 µl of protamine; stock 25 mg/ml in 1% Tween 20, Anachemia, Canada). After 24 hr, optical density (OD) of cultures was measured at 480 nm by Spectrophotometer (UNICCO Biotech Inc, Canada). Density of cultures was set to 1 OD. Broth was centrifuged at 6000 g (Beckman Instruments, USA) for 1 min to collect the cell pellet. Pellet was suspended in 1.0 ml cold PBS (pH 7.0). Cells were sonicated at setting of 10 for 1 min (Heat Systems-Ultrasonic’s Inc, USA) with an interval of 4-5 min on ice (3 times). Unbroken cells were separated by centrifugation at 5000 g for 8 min at 4 ^°^C. Supernatant was centrifuged at 100,000 g at 20 ^°^C for 30 min. Membrane pellets were collected and stored at – 80 ^°^C (Forma Scientific, USA) till needed. 

Pellets were removed from freezer and were suspended in 500 µl of 2% (w/v) Na-lauryl-sarcosine (Sigma) till completely dissolved. Solution was again ultra-centrifuged at 100,000 g at 20 ^°^C for 50 min. Collected pellets were resuspended in 200 µl of 2x sample buffer (Laemilli, 1970) with 10% β-mercaptoethanol (Sigma, USA). Samples were heated for 5 min in boiling water bath and samples were loaded on to SDS-PAGE (Bio Rad, USA) and stained with silver as reported previously (22).

***Standard linear regression curve***

Relative molecular weights of unknown proteins of *P. aeruginosa *were determined by standard linear regression curve (Bio Rad, USA). A standard curve of R_f_ values of standard proteins versus standard protein molecular weights was constructed to find out the best predicted values using the MS-Excel ver-2007 software. 

***Characterization of protamine on P. aeruginosa by electron microscopy***

Antimicrobial potential of protamine sulphate on *P. aeruginosa* cells was assessed by visualizing through electron micrographs taken at a resolution of 121,000 X. Effect of protamine, with and without stress, was determined as previously reported by Garduno and coworkers (22). Fresh culture was grown for 24 hr at 37 ^°^C in Nutrient broth and centrifuged as mentioned above. The pellets were resuspended and fixed in 500 µL of 25% glutaraldehyde solution (1 ml glutaraldehyde, (Merck, Germany), 9 ml of 100 mM sodium cocadylate (Sigma, Germany) pH 7.3 and 0.5% Alcian blue) and negatively stained in aqueous uranyl acetate, dehydrated in acetone, and embedded in epoxy (TAAB 812) resin. Thin sections of 100 nm were cut with microtome (LKB Instruments, Inc, USA) Thin sections were post stained with uranyl acetate-lead citrate and placed on 300 size mesh. The grids were air dried before they were electron micrographed with JEM 1230 Transmission Electron Microscope (JEOL Ltd, Japan) at 80 kV. Images were captured using a Hamamatsu ORCA-HR digital camera (Hamamatsu, Japan). All materials and reagents for EM were obtained from Marivac Ltd (Montreal, Canada).

## Results

Diabetes is a commonly reported metabolic disease, globally. In Pakistan, diabetic foot infection is a major complication in diabetes. In the present study, out of 386 patients, 350 (91%) patients were having various bacterial and fungal infections as indicated in Table 1. A higher prevalence up to (80%) was observed in male patients. This gender-based prevalence difference is probably due to the fact that males have more outdoor activities than females. *P. aeruginosa* was one leading isolated pathogen exhibiting antibiotic resistance as presented in Table 1. Among our isolates, *P. aeruginosa* shared 80 isolates (28.1%) of the total isolated pathogens. These isolates were resistant to different groups of antibiotics, such as gentamicin, cefotaxime, coamoxiclav, tazobactum and meropenem (Table 1).

Antimicrobial activity of Protamine was assessed against 20 clinical MDR *P. aeruginosa *from diabetic foot infections as well as revertant mutants of clinical *P. aeruginosa *3194 and 3248. MIC values for *P. aeruginosa *3194 and 3248 were also recorded, which was found to be 500 µg/ml of protamine. These isolates were subjected to maximum bactericidal concentration of protamine up to 2000 µg/ml.; these mutant isolates were reverted to normal when grown in protamine free medium.

**Figure 1.**
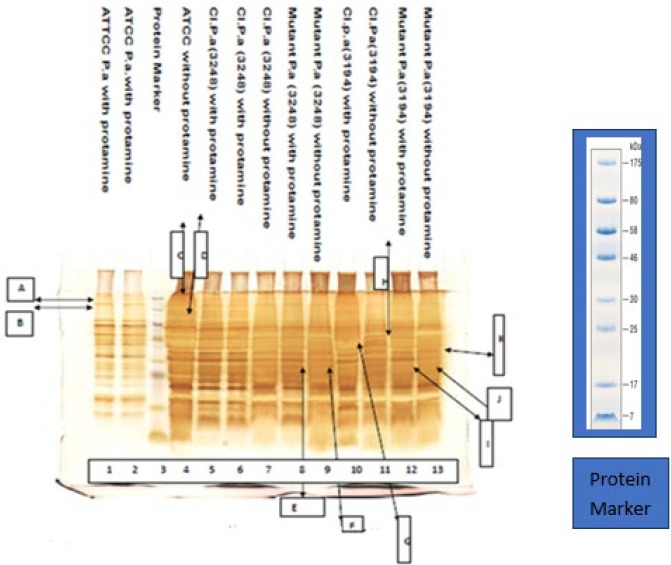
Effect of protamine on outer membrane proteins of *Pseudomonas aeruginosa*

Effect of protamine stress on outer proteins’ expression, using SDS PAGE, is presented Figure 1. In the presence of protamine, two extra bands i.e. A (175 kDa) and B (110 kDa) were identified (lanes 1 and 2, Figure 1), which were not observed in the absence of protamine (lane 4, Figure 1). Similarly, in the case of *P. aeruginosa *mutant 3248, two additional intense bands, band C (27 kDa) and band D (32 kDa) were identified in protamine induced expression (lane 8, Figure 1), while in absence of protamine, band D (32 kDa) was absent (lane 9). *P. aeruginosa *clinical isolate 3194, with addition of protamine band E of 50 kDa is absent while corresponding sample without protamine showed its expression in the gel (lane 11, Figure 1). In lane 12, *P. aeruginosa *mutant 3194, bands F (27 kDa) and G (32 kDa) were present in protamine stress condition, under non-stressed condition bands F (27 kDa) and G (32 kDa) were very weak i.e. corresponding proteins were found in low concentrations. We infer from these observations that different proteins were induced and/or suppressed after addition or in absence of Protamine (Figure 1).

**Figure 2:**
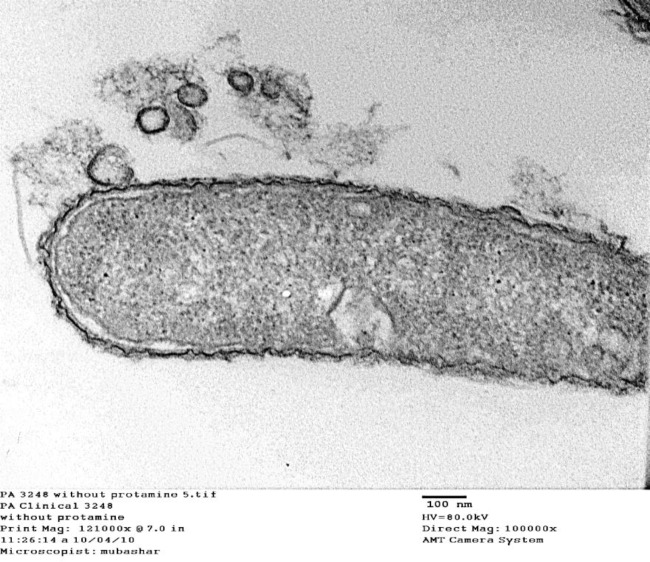
*Pseudomonas aeruginosa* without protamine (*P. aeruginosa* clinical isolate (3248) was used as standard to look insight the normal cellular structure (morphology). cell presented normal structure of outer membrane which was intact with vesicles)

**Figure 3:**
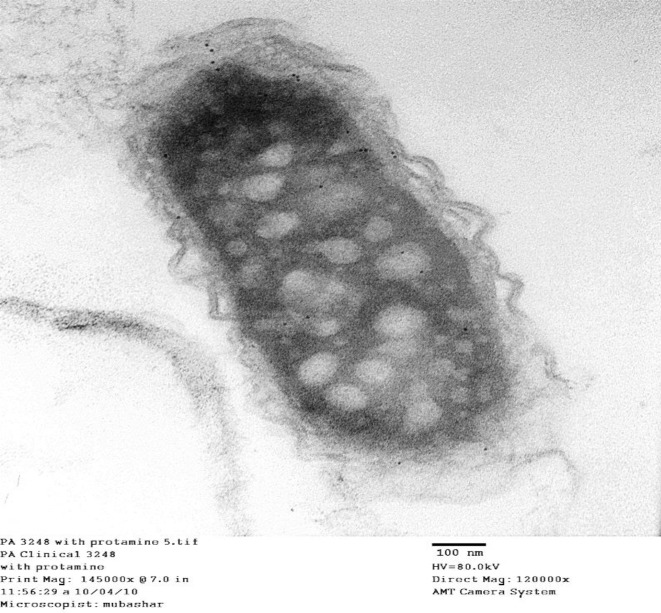
*Pseudomonas aeruginosa* with protamine (under stress condition of protamine, protamine was accumulated within the cytoplasm without damage to cell membrane or cell lysis. Protamine precipitation was evident within the cytoplasm)

**Figure 4:**
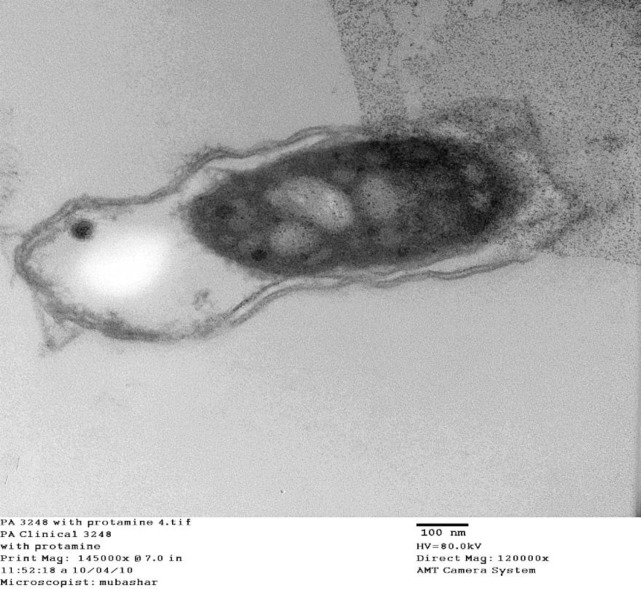
Effect of stress of protamine on *Pseudomonas aeruginosa *(under stress condition of protamine, cytoplasmic condensation of cell was observed with larger periplasmic space without cell lysis, Protamine precipitation was also evident within the cytoplasm)

Morphological and ultrastructural analysis of *P*. *aeruginosa *under nonstress and stressed conditions with protamine were analyzed further by transmission electron microscopy. Under non-stressed condition, clinical isolate* P*. *aeruginosa* 3248 showed normal outer membrane vesicular structures (Figure 2), while in the presence of protamine, condensation of cytoplasm was observed with enlarged periplasmic space (Figure 3). Protamine accumulation was also observed inside the cytoplasm along with partial damage to cell membrane with lysis (Figure 3). In some areas of cytoplasm, precipitation of protamine was also seen (Figure 4). These findings suggest that protamine induced changes at various outer membrane structures with disruption of cellular activities could be helpful to localize new antimicrobial targets.

## Discussion

Diabetes is a commonly reported metabolic disease, globally. In Pakistan, diabetic foot infection is a major complication in diabetics. Most of the time, pathogens involved in these infections are MDR isolates, which are difficult to treat. In the present study, pus specimens were collected from 386 diabetic patients with foot infection. Out of 386 samples, 350 (91%) patients yielded positive culture for various bacterial and fungal pathogens. Among bacterial isolates, Gram-negative *P. aeruginosa *shared 28.2% of the isolated pathogens. According to a study by Murugan and coworkers, *P. aeruginosa *was isolated in 18.9% of cases (23, 24). In a more recent report on diabetic foot infections, poly bacterial isolations yielded 19.2% cultures in Enterobacteriaceae family, with *P. aeruginosa *being 18.9% (25). In another study,* P. aeruginosa* was again found in 48% of the isolates as compared to *Staphylococcus aureus* (20%), *Staphylococcus epidermidis* (3%) and other Streptococci (3%) (26).

A recent study revealed that highly potent antibiotics, such as imipenem, meropenem, amikacin, piperacillin/tazobactam, were much more effective antibacterial agents against Gram negative isolates (25). In our data, high numbers of poly bacterial cultures were isolated with *P. aeruginosa* being the leading microbe. Most *P. aeruginosa* isolates were resistant to imipenam, meropenem, azotronam, ciprofloxacin, gentamycin, and piperacillin/tazobactam (Table 1). Bader (27) has already reported that disseminated infections cannot be treated with oral agents; rather they require more potent intravenous antibiotic infusions. Same findings were observed in our study and have previously been reported by Ozer and colleagues (25). All htese studies share very similar findings that our data also confirmed, which is presented here.

In the present study, antimicrobial potential of protamine was explored on MDR *P. aeruginosa*, a leading potential causative agent of diabetic foot infections. Our study data show that protamine, a new alternate antimicrobial agent from natural source (cationic antimicrobial peptide from fish salmon), is useful in treating *P. aeruginosa *infection, with 500 µg/ml. Action of protamine on different induced mutant strains revealed that they are effective in a range between 125 to 2000 mg/l, compared with 95 mg/l for the wild type strain. Protamine permeabilizes through cell membranes in both Gram-positive and Gram-negative bacteria (28). It is shown that protamine not only disturbs the membrane permeability but also influences energy transduction and nutrients’ amassing in bacterial cells (29). It may be speculated that protamine interacts with negatively charged bacterial covering electrostatically, resulting in leakage of potassium, ATP and other cellular components (28). In another report, protamine also showed good efficacy against biofilm developing *P. aeruginosa *(30). Literature on antimicrobial peptides, including protamine, have reported additional immune response activities, such as wound healing, recruitment of leucocytes and modulation of the inflammatory response (31) which is another benefit of its use.

Interestingly, 175 kDa and 110 kDa (*mexB, *an efflux pump) outer membrane proteins of *P. aeruginosa* isolates were absent without protamine as described by Schweizer (32). Our results show that protamine induced mutant profile also express 32 kDa protein (Triose phosphate isomerase). In the presence of protamine, 175 kDa and 110 kDa proteins were expressed. This signifies its inducible nature under stress. In case of clinical isolates, distinctive differences were observed in 50 kDa protein under these two different conditions. This protein, *opr M, *is also reported elsewhere, as *mex AB – opr M* complex in MDR efflux system of *P. aeruginosa *(33). Study on reverted mutants show that relative molecular weight of 27 kDa protein (petidyl-prolyl*cis*-*trans*isomerase PPIase) disappeared under protamine stress (34). This *mexAB – opr M* system, was also characterized by amplification technology in *P. aeruginosa *MDR isolates (Data not shown). We have observed the appearance of similar up-regulated results of *mexAB- oprM* proteins in clinical and mutant stains under protamine stress. This indicates the importance of protamine in lowering antibiotic resistance as documented by Broutin and workers (34). 

In our experiments on ultra-structure, *P*.* aeruginosa* (3248) clinical isolate showed normal outer vesicular membrane structure without protamine. These cells show condensation of the cytoplasm, enlarged periplasmic space and accumulation of protamine within the cytoplasm. We infer from our repeated observations on electron micrographs that there was no cell membrane lyses or any damage in these bacterial cells (Figures 3 and 4). Similar findings were also reported by Pink* et al. *(13) in *Salmonella typhimurium *cells where cells don’t lyse with the treatment of protamine. These results contradict the earlier studies where protamine stress was reported to disrupt and distend *P. aeruginosa *cells. These cells noticeably have damaged cytoplasmic membrane and granular cytoplasm (30, 36)*.*

Our data on protein profile and ultramicroscopic studies indicates otherwise (Figures 3 and 4). In another dissimilar note, no clumping or large holes were observed in *Listeria monocytogenes* and *Shewanella putrifaciens*, as documented by Johansen *et al.* (15). However, all these reports contain electron dense inclusions as observed by us in the present study. This electron dense translocation is unlikely to cross the phospholipid bilayer under the condition. Several reports suggested porin mediated antibiotic translocations, like those seen in protamine (12, 37, 38). In a more recent study, cationic selective barrel-like proteins (CSBPs) appear to mediate this transfer of protamine within the cytoplasm (13). 

**Table 1:** Antibiotic resistance pattern of *Pseudomonas aeruginosa* isolates (80) from diabetic foot infection

**Antimicrobial Group**	**Antimicrobial agent**	**No. of Isolates (%)**
		**Resistant Intermediate Sensitive**
Carbapenems	Imipenem	30 (37.5) 20 (25) 30(37.5)
	Meropenem	37 (46.25) 19(23.75) 24(30)
Cephalosporins	Ceftazidime	24 (30) 33(41.25) 23(28.75)
	Cefotaxime	35(43.75) 25(31.25) 20(25)
	Cefepime	28(35) 23 (28) 30(37)
Fluoroquinolones	Ciprofloxacin	25 (31.25) 27(33.75) 28(37)
	Levofloxacin	29(36.25) 26 (32.5) 25(31.25)
ß-lactamase inhibitors	Tazobactam	32(40) 28 (35) 20(25)
	Co amoxiclav	35 (43) 32 (39) 13(16)
Monobactams	Azetronam	31 (38.75) 11 (13.75) 38(47)
Aminoglycosides	Amikacin	29 (36.25) 15(18.75) 36( 45)
	Gentamicin	40(50) 21 (26.25) 19(23.75)

## Conclusion

Results of SDS-PAGE indicate that Protamine can suppress various outer membranes proteins while results of Transmission Electron Microscopy revealed suppressed cellular activities within *P. aeruginosa *which represent it as a good antimicrobial agent. Protamine sulphate may have the potential to be used as an alternate to antimicrobial agent, may be in the form of wound washing agent to reduce the microbial load. This hypothesis is needed to be further investigated by *in vivo* diabetic animal infection model study to have an insight into the Protamine effect on tissues by histopathology studies and to check cell cytotoxicity before its applications. 

## References

[1] Boulton AJ (2000). The diabetic foot: a global view. Diabetes/Metabolism Res. Rev.

[2] Raja NS (2007). Microbiology of diabetic foot infections in a teaching hospital in Malaysia: a retrospective study of 194 cases. J. Microbiol Immunol Infect.

[3] Siripornmongcolchai T, Chomvarin C, Chaicumpar K, Limpaiboon T, Wongkhum C (2002). Evaluation of different primers for detecting mecA gene by PCR in comparison with phenotypic methods for discrimination of methicillin-resistant Staphylococcus aureus. S. Asian J Trop Med Public Health..

[4] Frieri M, Kumar K, Boutin A (2017). Antibiotic resistance. J. Infect Public Health..

[5] Haynes A, Ruda F, Oliver J, Hamood AN, Griswold JA, Park PW, Rumbaugh KP (2005). Syndecan shedding contributes to Pseudomonas aeruginosa sepsis. Infect Immunity.

[6] Aspedon A, Groisman EA (1996). The antibacterial action of protamine: evidence for disruption of cytoplasmic membrane energization in Salmonella typhimurium. Microbiol.

[7] Gao B, Stieger B, Noé B, Fritschy JM, Meier PJ (1999). Localization of the organic anion transporting polypeptide 2 (Oatp2) in capillary endothelium and choroid plexus epithelium of rat brain. J. Histochem. Cytochem..

[8] Matsuzaki K, Yoneyama S, Fujii N, Miyajima K, Yamada KI, Kirino Y, Anzai K (1997). Membrane permeabilization mechanisms of a cyclic antimicrobial peptide, Tachyplesin I, and its linear analog. Biochem.

[9] Wu M, Maier E, Benz R, Hancock RE (1999). Mechanism of interaction of different classes of cationic antimicrobial peptides with planar bilayers and with the cytoplasmic membrane of Escherichia coli. Biochem.

[10] Hancock RE (1997). Antibacterial peptides and the outer membranes of gram-negative bacilli. J Med Microbiol.

[11] Hancock RE (1997). Peptide antibiotics. The Lancet.

[12] Pink DA, Hasan FM, Quinn BE, Winterhalter M, Mohan M, Gill TA (2014). Interaction of protamine with gram-negative bacteria membranes: possible alternative mechanisms of internalization in Escherichia coli, Salmonella typhimurium and Pseudomonas aeruginosa. J. Peptide Sci.

[13] Pink D, Hansen LT, Gill T, Quinn B, Jericho M, Beveridge T (2003). Divalent calcium ions inhibit the penetration of protamine through the polysaccharide brush of the outer membrane of Gram-negative bacteria. Langmuir.

[14] Hansen LT, Gill TA (2000). Solubility and antimicrobial efficacy of protamine on Listeria monocytogenes and Escherichia coli as influenced by pH. J Appl Microbiol.

[15] Johansen C, Verheul A, Gram L, Gill T, Abee T (1997). Protamine-induced permeabilization of cell envelopes of gram-positive and gram-negative bacteria. Appl. Environ. Microbiol.

[16] Johansen C, Gill T, Gram L (1996). Changes in cell morphology of Listeria monocytogenes and Shewanella putrefaciens resulting from the action of protamine. Appl Environ Microbiol.

[17] Johansen C, Gill T, Gram L (1995). Antibacterial effect of protamine assayed by impedimetry. J Appl Bacteriol.

[18] Uyttendaele M, Debevere J (1994). Evaluation of the antimicrobial activity of protamine. Food Microbiol.

[19] Rosa-Fraile M, Camacho-Muñoz E, Rodríguez-Granger J, Liébana-Martos C (2005). Specimen storage in transport medium and detection of group B Streptococci by culture. J Clin Microbiol.

[20] Gadepalli R, Dhawan B, Sreenivas V, Kapil A, Ammini AC, Chaudhry RA (2006). Clinico-Microbiological study of diabetic foot ulcers in an Indian tertiary care hospital. Diabetes Care.

[21] Willcox MD, Hume EB, Aliwarga Y, Kumar N, Cole N (2008). A novel cationic-peptide coating for the prevention of microbial colonization on contact lenses. J Appl Microbiol.

[22] Garduno RA, Garduno E, Hiltz M, Hoffman PS (2002). Intracellular growth of Legionella pneumophila gives rise to a differentiated form dissimilar to stationary-phase forms. Infect. Immunity.

[23] Murugan S, Mani KR, Uma Devi P (2008). Prevalence of methicillin resistant Staphylococcus aureus among diabetes patients with foot ulcers and their antimicrobial susceptibility pattern. J Clin Diagn Res.

[24] Ozer B, Kalaci A, Semerci E, Duran N, Davul S, Yanat AN (2010). Infections and aerobic bacterial pathogens in diabetic foot. Afr. J Microbiol Res.

[25] Khoharo HK, Ansari S, Qureshi F (2009). Diabetic foot ulcers; common isolated pathogens and in vitro antimicrobial activity. Profess. Med J Quart.

[26] Olid AS, Solà I, Barajas-Nava LA, Gianneo OD, Cosp XB, Lipsky BA ( 2015). Systemic antibiotics for treating diabetic foot infections. Cochrane Database of Systematic Reviews.

[27] Pränting M, Andersson DI (2010). Mechanisms and physiological effects of protamine resistance in Salmonella enterica serovar typhimurium LT2. J Antimicrob Chemother.

[28] Koo SP, Bayer AS, Yeaman MR (2001). Diversity in antistaphylococcal mechanisms among membrane-targeting antimicrobial peptides. Infect. Immunity.

[29] Soboh F, Khoury AE, Zamboni AC, Davidson D, Mittelman MW (1995). Effects of Ciprofloxacin and Protamine sulfate combinations against catheter-associated Pseudomonas aeruginosa biofilms. Antimicrob. agents Chemother.

[30] Himly M, Mills-Goodlet R, Geppert M, Duschl A (2017). Nanomaterials in the Context of Type 2 immune Responses—Fears and Potentials. Frontiers Immunol.

[31] Schweizer HP (2003). Efflux as a mechanism of resistance to antimicrobials in Pseudomonas aeruginosa and related bacteria: unanswered questions. Genet Mol Res.

[32] Sun J, Deng Z, Yan A (2014). Bacterial multidrug efflux pumps: mechanisms, physiology and pharmacological exploitations. Biochem. Biophys. Res. Commun..

[33] Mohan M (2010). Effects of Protamine on Pseudomonas aeruginosa cell envelope components: surface remodeling [M Sc dissertation]. Dalhousie University, Halifax Canada.

[34] Broutin I, Benabdelhak H, Moreel X, Lascombe MB, Lerouge D, Ducruix A (2005). Expression, purification, crystallization and preliminary X-ray studies of the outer membrane efflux proteins OprM and OprN from Pseudomonas aeruginosa. Acta Crystallograph Section F: Struct Biol Crystal Commun.

[35] MacMillan WG, Hibbitt KG (1969). The effect of antimicrobial proteins on the fine structure of Staphylococcus aureus. Microbiol.

[36] Mahendran KR, Kreir M, Weingart H, Fertig N, Winterhalter M (2010). Permeation of antibiotics through Escherichia coli OmpF and OmpC porins: screening for influx on a single-molecule level. J Biomol Screening.

[37] Danelon C, Nestorovich EM, Winterhalter M, Ceccarelli M, Bezrukov SM (2006). Interaction of zwitterionic penicillins with the OmpF channel facilitates their translocation. Biophys J.

[38] Aspedon A, Groisman EA (1996). The antibacterial action of protamine: evidence for disruption of cytoplasmic membrane energization in Salmonella typhimurium. Microbiology.

